# Extraarticular Pretibial Synovial Cyst After Arthroscopic Anterior Cruciate Ligament Reconstruction: A Case Report

**DOI:** 10.7759/cureus.26139

**Published:** 2022-06-21

**Authors:** Rheema Alfadhil, Ibrahim Alshaygy, Fawzi Aljassir

**Affiliations:** 1 Department of Orthopaedics, King Saud University, Riyadh, SAU

**Keywords:** anterior cruciate ligament (acl), tibial tunnel cyst, cyst, complication, anterior cruciate ligament reconstruction, pretibial cyst

## Abstract

Arthroscopic reconstruction of the anterior cruciate ligament has been modernized through new materials and novel surgical techniques. The usage of bioabsorbable screws for tibial fixation may potentially lead to complications, such as formation of a tibial tunnel or pretibial cysts. This is a relatively rare complication, but it has been described in the literature. The pathomechanism of cyst formation, however, still remains poorly understood. In this case report, we present a case of a healthy 23-year-old gentleman who had left tibia vara, which was treated surgically with proximal tibia corrective osteotomy with plate and screw fixation and subsequent hardware removal. Later in his life, he injured his anterior cruciate ligament, which required arthroscopic reconstruction. Years after, he developed a pretibial synovial cyst, which was visualized on magnetic resonance imaging. We reviewed previously published cases with similar presentations to help describe the possible etiology of intraosseous (tibial tunnel) cysts.

## Introduction

Arthroscopic reconstruction of the anterior cruciate ligament (ACL) has been modernized through new materials and novel surgical techniques. The usage of bioabsorbable screws for tibial fixation may lead to potential complications, such as formation of a tibial tunnel or pretibial cysts [1]. This is a relatively rare complication described in the literature. The pathomechanism of cyst formation, however, remains poorly understood [2]. The occurrence of tibial tunnel cysts, including pretibial cysts, after ACL reconstruction (ACLR) is infrequent, with only a limited number of cases reported in the literature. We present a case of a healthy 23-year-old gentleman who had left tibia vara, which was treated surgically with proximal tibia corrective osteotomy with plate and screw fixation and subsequent hardware removal. Later in his life, he injured his ACL, which required arthroscopic reconstruction. Years after, he developed a pretibial synovial cyst, which was visualized on magnetic resonance imaging. In addition, we reviewed previously published cases with similar presentations to help describe the possible etiology of intraosseous (tibial tunnel) cysts.

## Case presentation

A 23-year-old gentleman sustained a twisting injury to his left knee in 2017. He had a history of left tibia vara treated with a proximal tibial osteotomy (2007) with subsequent hardware removal (2010). Examination revealed positive Lachman’s test and laxity, intact posterior drawer test, and stable collateral ligaments. MRI confirmed an anterior cruciate ligament tear. In 2018, he underwent arthroscopic left knee ACLR with hybrid grafting (gracilis autograft and semitendinosus allograft), as semitendinosus adhered to the medial collateral ligament. Proximally adjustable end button and distal fixation were obtained using a bioabsorbable poly-D-L-lactide (PDLLA) 10 mm interference screw (Arthrex, USA). A stable knee was obtained after fixation. The two-week postoperative visit revealed a clean healing wound.

**Figure 1:**
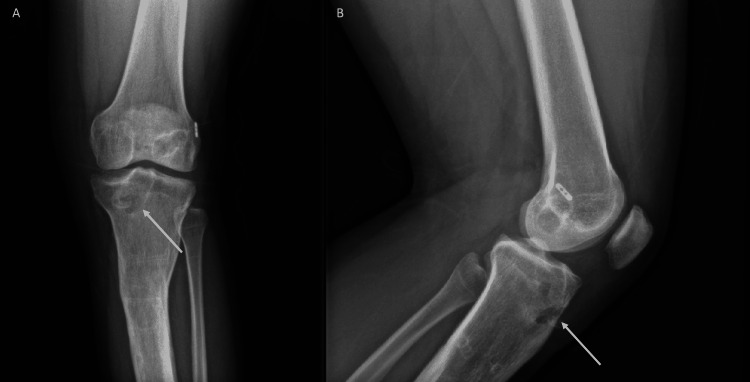
Anteroposterior and lateral X-rays of the pretibial cystic lesion prior to curettage and grafting. A) Left knee anteroposterior view showing the pretibial cyst; B) left knee lateral view showing the pretibial cyst.

In 2020, he presented with pain, redness, and swelling of the overlying skin around his previous arthroscopic surgical site. We suspected cellulitis and ordered a septic workup, X-ray, MRI, and prescribed amoxicillin with clavulanic acid in addition to analgesia. His septic workup came back negative and his X-ray showed a pretibial cystic lesion over the proximal tibia (Figure 1).

**Figure 2:**
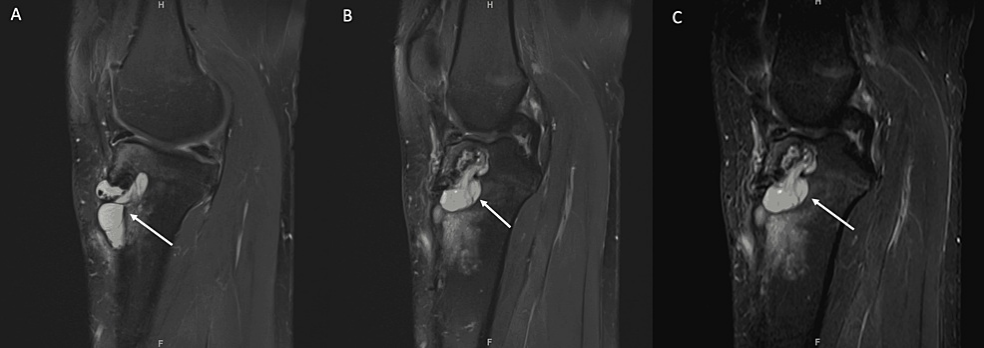
Sagittal knee MRI scans of the pretibial cyst showing its communication with the tibial tunnel. A) Sagittal knee MRI scan (proton density fat-saturated (PD FS)); B) sagittal knee MRI scan (PD FS); C) sagittal knee MRI scan (short T1 inversion recovery (STIR)).

His MRI revealed a bright lesion with graft mucoid degeneration and tibial tunnel widening. Within the tibial tunnel lied a ganglion cyst communicating with the pretibial area. Surrounding the tunnel, significant bone marrow signal alterations involving the proximal tibial epiphysis and metaphysis were noted. The posterior cruciate ligament was intact. There was a complex tear involving the posterior aspect of the medial meniscus (Figure 2).

Inflammatory markers were normal, making osteomyelitis unlikely.

**Figure 3:**
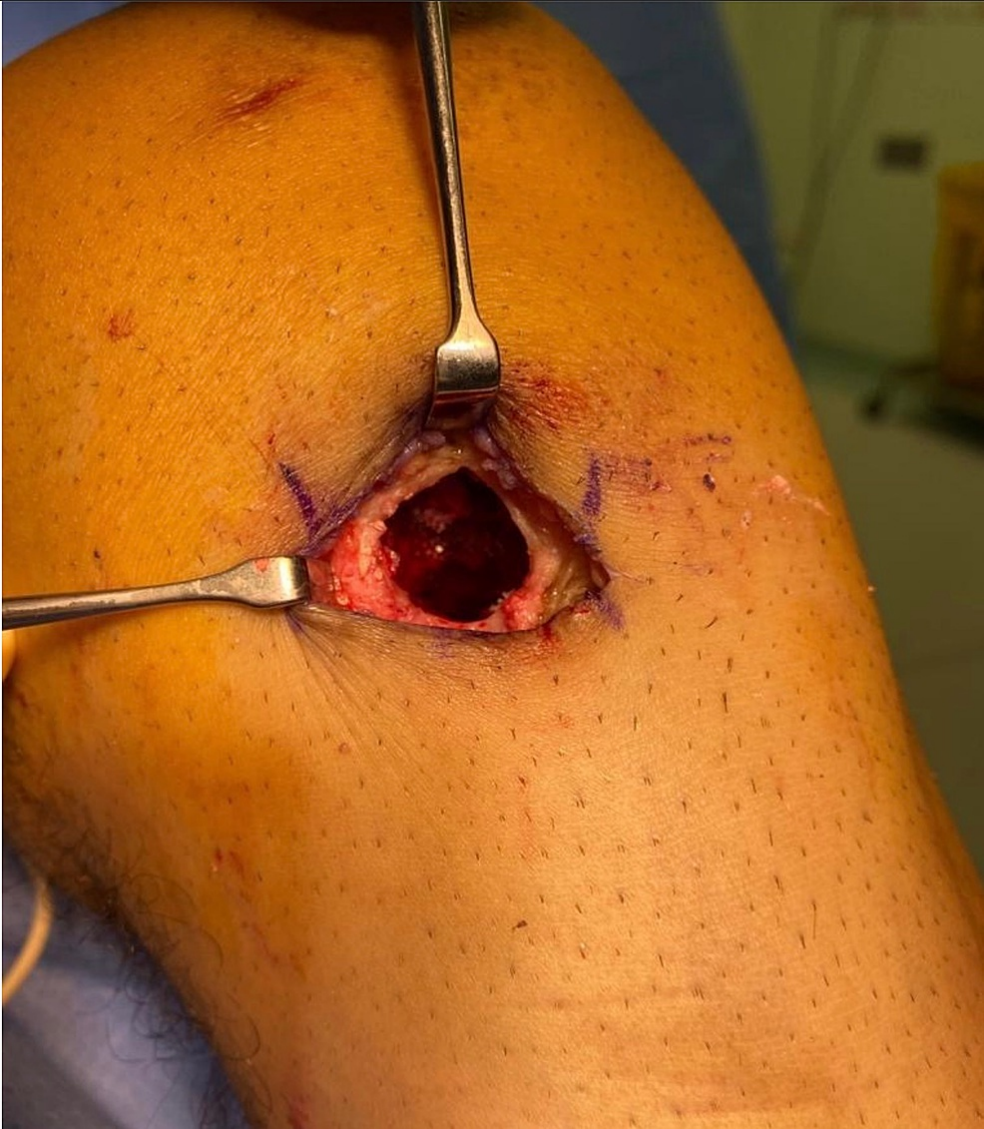
Intraoperative image of the cyst cavity.

He was admitted the following day for cyst curettage and bone grafting. Intraoperatively, ACL testing was done showing an intact reconstructed ligament. The previous incision was utilized and clear fluid with remaining screw particles was produced from the cyst. It did not communicate with the joint despite the MRI finding (Figure 3).

Tissue samples were sent for histopathology and cultures. Debridement was done and the bony defect was impacted with cancellous bone chips. Closure was done. Wound and tissue cultures were negative. He was advised to practice non-weight-bearing, provided with analgesia and antibiotics, and discharged. The histology result slowed vascularized fibroadipose tissue with focal synovial lining, which is consistent with synovial cysts. It showed foci of chronic inflammation and bone fragments. The sample stained positive for CD163. Pan-cytokeratin (pan-CK) was negative in the lining cells.

Upon wound inspection, swelling and oozing of serosanguinous fluid were noted therefore, manual evacuation was performed. Samples were not sent for microbiology.

**Figure 4:**
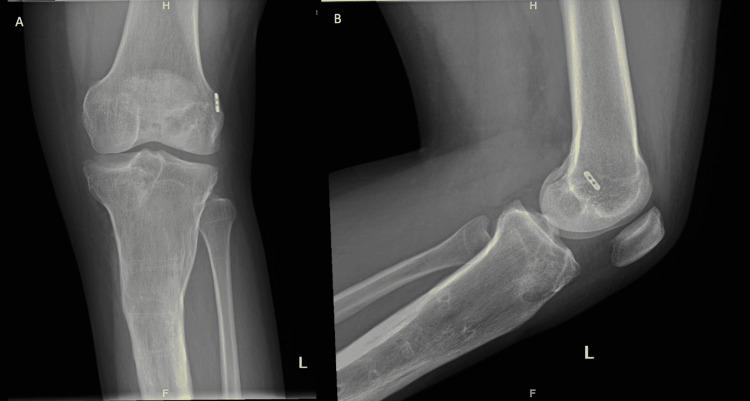
Anteroposterior and lateral X-ray of the pretibial cystic lesion 2 weeks after curettage and grafting. A) Anteroposterior X-ray of the pretibial cystic lesion 2 weeks after curettage and grafting; B) lateral X-ray of the pretibial cystic lesion 2 weeks after curettage and grafting.

He was given a close follow-up with imaging and prescribed additional antibiotics (Figure 4).

During such, pain improved and the wound stopped producing discharge. He was allowed to bear weight as tolerated. He was allowed to progress to full weight-bearing six weeks after the procedure, and since then he has been doing well with a healed wound and good range of motion (ROM) and mobilization. We were not able to secure a clinical picture of his healed wound at the time. He complains of occasional mild anterior knee pain over the tibial tubercle and is still being followed up in the clinic. No additional radiological investigations were done to objectively assess the healing of the lesion. He’s currently continuing physical therapy, strengthening exercises, and avoiding vigorous activity. The patient’s informed consent was sought and granted.

## Discussion

Arthroscopic reconstruction of the ACL has been modernized through novel materials and surgical techniques. Usage of bioabsorbable screws for tibial fixation may potentially lead to complications, such as tibial tunnel or pretibial cysts, mostly occurring 1-5 years postoperatively [1,3-5]. In published reports, there was no association between cyst formation and graft failure, instability, or loosening [3].

Synovial cysts are fluid-filled juxta-articular cysts lined by synovial cells that may or may not communicate with the joint fluid [6]. Some suggested that revision surgeries may increase their occurrence due to the presence of multiple tunnels [7]. Cysts are typically asymptomatic and are incidentally discovered by MRI, but occasionally may cause swelling, limited ROM, or pain from compression against the surrounding tissue [3,4].

On MRI, cysts appear as well-defined uni/multilocular cystic masses [6]. There may be tunnel widening, periosteal reaction around the device, pressure erosions and remodeling around the tunnel, or reactive marrow edema [3]. These are best appreciated on fluid-sensitive sequences (fat-suppressed T2 and short tau inversion recovery) [3]. Recurrence is rare and may be due to incomplete resection or curettage [8,9]. Overall, most patients experienced full relief after resection.

There were a variety of graft materials and several tibial fixation devices utilized [2]. Despite this, the phenomenon of tibial cysts after ACL reconstruction remains poorly understood, making it difficult to establish a single etiology for their emergence [1]. Several hypotheses have explored the reasons for their formation, dividing cysts into communicating or non-communicating tunnel cyst formation [3].

The occurrence of non-communicating cysts has been most prominently linked to a bony reaction to fixation devices (which may cause communicating or non-communicating) and breakdown of bioabsorbable interference screws [1,2,10]. Several authors have related cyst formation to screw breakdown, causing fluid collections. However, not all collections mature into cysts [1]. It is important to note that finding an isolated small fluid collection in the osseous tunnel after ACL reconstruction is considered a normal finding and is expected to resolve within 18 months [3,4]. This may be attributed to using cannulated screws [1,4]. Gonzalez-Lomas et al. used nanocrystalline poly-L-lactic acid (PLLA) bioabsorbable screws in arthroscopic ACL reconstruction and proposed that a sterile foreign body reaction occurred due to screw breakdown which caused cyst formation [1]. PLLA screw breakdown has been described to possibly incite a foreign body reaction leading to development of cysts [1,10]. This was supported by the absence of recurrence after removal of screw materials [1].

Communication of cysts to the joint may not be easily demonstrated, therefore, the presence of chondroitin sulfate in cyst analysis may indicate that the fluid came from the joint [3]. Communicating cysts have been proposed to occur after the use of bioabsorbable interference screws due to a foreign body reaction [2]. This and screw degradation would lead to the reopening of water channels, allowing communication of intraarticular fluid with the extraarticular cyst [2].

Tsuda et al. theorized that cyst occurrence was due to leakage of articular fluid through the tibial tunnel [2]. This was said to be caused by graft-tunnel mismatch, intraosseous graft necrosis, screw breakdown, eccentric placement of the graft in the tunnel, and lack of osteointegration of the graft into the tunnel [2]. Victoroff et al. suggested that tunnel enlargement could be attributable to incomplete allograft incorporation within the tibial tunnel and residual graft necrosis, allowing synovial fluid to communicate with the pretibial area leading to pretibial cysts [11].

Simonian et al. described incomplete autograft incorporation [12]. When excess knee joint fluid is compressed by body weight between the bones, this may lead to the fluid escaping through the weakest point in the joint capsule, in this case as a protrusion through the tibial tunnel. This occurs because full osteointegration needs several years to occur [13]. Also explains why tibial tunnel and subcutaneous pretibial area are the most common areas affected [13]. Victoroff et al. supported this theory but using an Achilles tendon allograft. This indicates that relatively little is known about graft remodeling and integration and possible immunological responses [11].

Ahn et al. described an incidental cyst after posterior cruciate ligament reconstruction. They theorized that fluid leakage and cyst formation was due to failed osteointegration of the graft into the tibial tunnel [14]. It was highlighted that flexor tendon grafts do not have a bone block that consolidates the tunnel, possibly leading to cyst formation [4].

Fewer studies believed the occurrence of cysts may be due to using nonabsorbable sutures, drilling oversized tunnels, or tunnel-graft size mismatch [14]. This is less likely, as cysts tend to occur years after the surgery. If those were the causes, patients would likely present sooner [12].

Bioabsorbable screws fragment once they are hydrolyzed, producing a phagocytic reaction by macrophages, which may elicit an inflammatory foreign body reaction [1,13]. Degradation rates differ between bioabsorbable screw materials. For instance, polyglyconate screws degrade rapidly, whereas PLLA implants have a slower degradation rate, potentially eliciting a delayed nonspecific foreign body reaction [1].

We believe the etiology is multifactorial. In our patient, several causes can be attributed to the formation of the cyst. We used a bio-absorbable screw, and a hybrid graft (autograft and allograft). Both have been documented to carry the rare potential risk of cystic formation years after the procedure. The MRI showed a communicating cyst, in contrast to intraoperative assessment, as well as a significant tunnel widening, favoring the theory of an inflammatory reaction due to the screw breakdown, as this takes years to occur. In our case, the cyst presented 2 years postoperatively. Furthermore, histologic results of chronic inflammatory signs support the theory of a foreign body reaction occurring while the screw was breaking down. Remaining screw particles were found intraoperatively, possibly indicating that the screw was undergoing resorption at the time of surgery. Second, lack of complete osteointegration of the graft into the tunnel could lead to the MRI findings. This can be supported by using a hybrid reconstruction, as we know little about how allografts integrate and whether they may induce an immunological reaction, which may explain the chronic inflammatory changes seen on histology. Third, this could lie in the patient’s history of having a corrective osteotomy years earlier, allowing for the focal bone to become weaker, owing to the occurrence of the cyst in the same area of the proximal tibia. The latter theory does not explain the chronic inflammatory reaction, but it does explain the cyst lining being that of a synovial cyst. To add, we have not taken a sample of the femoral aspect of the tunnel for biopsy, to concur whether or not there were chronic inflammatory changes there as well. Therefore, we are unable to attribute this complication to a single theory.

Despite the perceivable advantages of bioabsorbable screws, awareness of their complications is essential. Given the infrequency of cyst formation compared to the number of ACL reconstructions being performed with bioabsorbable screws, it raises the possibility of a patient-related factor. It was proposed that this may be due to a sensitivity to larger foreign bodies [1].

There are several limitations. First, as a case report, we are unaware of other cases that were not evaluated further. Second, we used a hybrid graft and have no other cases to infer from in terms of rates of occurrence in such grafts. Moreover, our sample was only investigated by light microscopy and immunohistochemistry, without biochemical analysis of the fluid. Despite this, up to our current knowledge, no cases have been published from Saudi Arabia.

Due to the limited number of published cases and poor understanding of the mechanism of cyst development and the long-term outcomes, and the many variables mentioned, it is challenging to narrow down the direction of prospective studies. It will be difficult to find an adequate sample size, due to rare occurrences and lack of symptoms. Areas worthy of addressing are the different surgical techniques, graft preparations, and types of grafts utilized. We would also recommend addressing individual-to-individual variations in biodegradation and immunological mechanisms.

## Conclusions

The occurrence of the tibial tunnel and pretibial cysts after ACLR is infrequent, with a limited number of cases reported in the literature. Our current case is interesting as our patient had a history of undergoing tibial corrective osteotomy for tibia vara years before undergoing ACLR. It is likely that the etiology behind his developing this pretibial cyst is multifactorial, as several causes can be attributed to the formation of this cyst, including the usage of a bioabsorbable screw and hybrid graft, the possible inflammatory reaction due to the screw breakdown, the possibility of incomplete osteointegration of the graft, or the possibility of a patient-related factor as he had a corrective osteotomy years earlier in the same location of the proximal tibia. We highlight the importance of the awareness of the potential complications of bioabsorbable screws, despite their perceivable advantages.
